# Analysis of *MIR155HG* gene polymorphisms and ulcerative colitis susceptibility in the Chinese Han Population

**DOI:** 10.1016/j.ncrna.2026.01.012

**Published:** 2026-02-03

**Authors:** Chao Xu, Beibei Zhou, Fan Bai, Hang Sun, Cuiping Zhang, Zhenlun Wu, Jiachuan Hou, Jinjing Xie, Yanbin Wei, Libin Pan, Ruiqi Yang, Hongjie Dong, Guihua Zhao, Jingyu Yang, Jianwei Zhou, Ruili Wu, Kun Yin

**Affiliations:** a Digestive Disease Hospital of Shandong First Medical University, Shandong Institute of Parasitic Diseases, Shandong First Medical University & Shandong Academy of Medical Sciences, Jining, 272033, China; b School of Public Health, Shandong First Medical University & Shandong Academy of Medical Sciences, Jinan, 271016, China; c Clinical Laboratory, Affiliated Hospital of Jining Medical University, Jining, 272029, China; d Yantai Affiliated Hospital of Binzhou Medical University, Yantai, 264100, China; e Jiaxiang County People's Hospital, Jining, 272400, China; f Jining Rencheng District People's Hospital, Jining, 272000, China

**Keywords:** Ulcerative colitis, *MIR155HG*, polymorphisms, Chinese Han population, Case-control study

## Abstract

Aberrant expression of miR-155 has been implicated in the pathogenesis of inflammatory bowel disease (IBD), where its dysregulation may contribute to impaired intestinal barrier integrity and sustained inflammation. However, the precise role of miR-155 in the development of ulcerative colitis (UC) remains incompletely understood. This case-control study aimed to evaluate the association between polymorphisms of *MIR155HG* gene and susceptibility to UC in a Chinese Han population. The study included 84 UC patients and 216 matched healthy controls. Four single nucleotide polymorphisms (SNPs) of *MIR155HG* (rs1893650, rs2282471, rs2829803, and rs2829806) were genotyped using the Sequenom MassARRAY platform. Function prediction of SNPs were conducted using RNAfold databases. We observed *MIR155HG* rs2282471 polymorphism was significantly associated with a reduced risk of UC. Specifically, the minor T allele, homozygous TT genotype, and the recessive genetic model (TT vs CT + CC) of rs2282471 conferred protective effect to the disease. The stratified analysis indicated this protective effect was more pronounced in male patients. Furthermore, haplotype analysis showed a strong linkage disequilibrium among the four SNPs and identified a specific haplotype (TGTT) that was also associated with decreased UC risk. No significant correlations were observed between four SNPs and clinical features such as disease severity or lesion location. Structure prediction suggested that rs2282471 may influence the secondary structure of miR-155. These findings provided the first evidence that the *MIR155HG* rs2282471 polymorphism were associated with decreased UC susceptibility in the Chinese Han population, suggesting *MIR155HG* might be a predictive biomarker for risk of UC.

## 1 Introduction

Inflammatory bowel diseases (IBDs) are a group of chronic inflammatory disorders affecting the gastrointestinal tract, which significantly impact the quality of life of patients and have become a growing global health concern [1]. The incidence and prevalence of IBD have been steadily increasing worldwide, particularly in industrialized countries, with a notable upward trend also observed in developing countries [2]. IBD comprises two main subtypes: ulcerative colitis (UC) and Crohn's disease. UC is a chronic, non-specific form of IBD that characterized by continuous mucosal inflammation extending from the rectum to the proximal colon, and it typically follows a relapsing and remitting course [3]. The symptoms not only disrupt normal digestive function of patients but also cause severe physical and mental distress, often leading to long-term burdensome complications. Elucidating the underlying mechanisms of UC can provide valuable insights for developing of targeted therapeutic strategies, improving the treatment efficacy, and ultimately enhancing the patients' quality of life. However, the exact pathogenesis of UC remains complex and incompletely understood, of which the pathogenesis involves complex interactions among genetic background, immune responses, environmental variables, and intestinal flora [4,5].

MicroRNAs (miRNAs) are a class of small non-coding RNAs with a length of 21–25 nucleotides. They bind to the 3′ untranslated region (3′UTR) of target RNAs through complementary pairing, thereby degrading the target RNAs or inhibiting their transcription and translation [6]. In this way, miRNAs participate in the post-transcriptional regulation of genes, which regulate various cell activities and involve in the development of immune system [7]. MiR-155, encoded by the host gene *MIR155HG*, is one of the first identified miRNAs and has been the focus of extensive research due to its important role in immune-related functions [8]. Previous studies have demonstrated that expression of miR-155 was abnormal in inflammatory autoimmune disorders, and functional analysis have further indicated that this molecule participated in the pathogenesis of these diseases by regulating several signaling pathways [9,10]. These findings suggested that genetic variations in the *MIR155HG* gene might have a significant impact on the susceptibility to these diseases. Nevertheless, the precise role of miR-155 in IBD has not been fully elucidated.

In China, the landscape of IBD had undergone a significant transformation over the past three decades, with both its incidence and prevalence rising at an unprecedented rate—a trend that closely parallels the nation's rapid economic development and profound shifts in lifestyle patterns, which severely affected patients' quality of life and posed further challenges to China's healthcare system [11,12]. Although miR-155 has been shown to be highly expressed in the inflamed intestinal mucosa of IBD patients, where it contributed to the release of pro-inflammatory cytokines, the relationship between its genetic polymorphisms and susceptibility to UC remains unclear, especially among the Chinese Han population. Therefore, in this study, we conducted a case-control study among a Chinese Han population to explore the association between *MIR155HG* polymorphisms and risk of UC, aiming to provide a basis for further clarifying the genetic and immunological pathogenesis of UC.

## 2 Materials and methods

### 2.1 Study population

Between June 2023 and October 2024, patients with UC were recruited from individuals who visited the Digestive Disease Hospital of Shandong First Medical University (Jining, China), Jiaxiang County People's Hospital (Jining, China), and Rencheng District People's Hospital (Jining, China). The inclusion criteria were as follows: (1) patients fulfilled the clinical, endoscopic, radiological, and histological diagnosis criterion of UC according to Chinese consensus on diagnosis and treatment in inflammatory bowel disease (2018, Beijing) guideline [13]; (2) patients had no other digestive diseases, autoimmune diseases, inherited diseases, chronic inflammatory diseases, viral infectious diseases, or malignancies. A questionnaire was used to collect information on age, gender, lesion location, clinical symptoms, and history of smoking and alcohol consumption. The lesion location of UC was classified according to the Montreal classification, while disease severity was assessed through the Truelove and Witts' criteria [14,15]. A total of 84 eligible UC patients were enrolled in this study. During the same period, a total of 216 age- and sex-matched healthy controls were recruited from individuals undergoing routine physical examinations. The controls had no history of IBD, chronic diseases, hereditary diseases, infectious diseases, or autoimmune diseases. None of the participants were blood relatives.

### 2.2 Ethical approval

This study was reviewed and approved by the Ethics Committee of the Digestive Disease Hospital of Shandong First Medical University (approval number: DDH-KY2023003). Informed consent was obtained from each participant or the guardians of minor subjects prior to study initiation.

### 2.3 Blood sampling and DNA extraction

Approximately 2 mL peripheral venous blood was collected from UC patients (before receiving any treatment) and health controls, and stored in Vacutainer tubes containing the anticoagulant EDTA (BD Biosciences, Franklin Lakes, NJ, USA). Genomic DNA was extracted from the blood samples using a Blood DNA Mini Kit (Omega Bio-tek, Inc., Doraville, USA), according to the manufacturer's instructions. The DNA yield and quality were evaluated using a NanoDrop 2000 spectrophotometer (Thermo Fisher Scientific, Waltham, MA, USA), and then stored at −20 °C until use.

### 2.4 SNP selection

Four candidate single nucleotide polymorphisms (SNPs) rs1893650, rs2282471, rs2829803, and rs2829806 of *MIR155HG* were selected. These SNPs have previously been reported to be associated with susceptibility to multiple human autoimmune diseases [[16], [17], [18], [19], [20]]. All candidate SNPs were in accordance with the quality control standards, with minor allele frequencies (MAF) > 0.05 in the Han Chinese in Beijing (CHB) database referenced from the 1000 Genomes Project (https://www.internationalgenome.org/). Linkage disequilibrium (LD) was further analyzed using the Haploview software (version 4.2).

### 2.5 Genotyping

Forward, reverse, and extension primers for PCR amplification were designed using the Assay Design software (Sequenom Inc, San Diego, CA, USA) (Table 1). Genotyping of candidate SNPs was performed using the iPLEX Assay and MassARRAY system (Sequenom Inc., San Diego, CA, USA) through chip-based matrix-assisted laser desorption/ionization time of flight mass spectrometry (MALDI-TOF MS). Data were analyzed using the MassARRAY Typer software (Sequenom Inc., San Diego, CA, USA).

**Table 1:** The basic information and primers used for candidate SNPs of *MIR155HG*.

SNP	Chromosome position	Location	Alleles	Forward primers	Reverse primers	Extension primers
**rs1893650**	26940815	Intron variant	T/C	ACGTTGGATGCCTGCTTCTGCTTATTCCAC	ACGTTGGATGGTGCTTTCGCTTTTCTGATG	tcttCTTATTCCACAGAATGAG
**rs2282471**	26956748	Intergenic variant	T/C	ACGTTGGATGGAGATGTCAAAAATTGAAAGG	ACGTTGGATGCCTGTACCTGTATCTAGGTC	ATTGAAAGGATCGCCTA
**rs2829803**	26948310	Intergenic variant	G/A	ACGTTGGATGGTGTACCCAAATGCTCTTC	ACGTTGGATGCCTTCCTGCTGTTACAATAA	AAATAAGGCATTTCC
**rs2829806**	26960189	Intron variant	G/T	ACGTTGGATGGTCCTCATTAGGACTTCCTC	ACGTTGGATGGTGATGCTTAATGTGCCTGC	CACAATGCCACAAAC

### 2.6 In silico analysis

Changes in secondary structure and minimum free energy (MFE) resulting from SNP effects were predicted using RNAfold (http://rna.tbi.univie.ac.at/cgi-bin/RNAWebSuite/RNAfold.cgi) [21]. Alterations in secondary structure and MFE may affect the binding affinity of microRNAs. Free energy denotes the amount of energy that required to drive structural transformation, where a lower value corresponds to a greater structural stability.

### 2.7 Statistical analysis

All statistical analyses were conducted using SPSS version 22.0 (SPSS Inc., Chicago, IL, USA). The Hardy-Weinberg equilibrium (HWE) was assessed any deviation of genotypic frequency with the goodness-of-fit test for *MIR155HG* SNPs in the healthy control group. The frequencies of alleles, genotypes, as well as dominant and recessive genetic models, were compared between the case and control groups using Pearson's Chi-square test or Fisher's exact test. The odds ratio (OR), 95 % confidence interval (CI), and *P*-values were calculated through the unconditional logistic regression analysis. Multiple comparisons in the SNP analysis were adjusted using the Holm-Bonferroni method, which was employed to avoid false-positive associations caused by multiple hypothesis testing. The LD and haplotype construction were performed using Haploview software (version 4.2). A *P*-value <0.05 was considered to be statistically significant.

## 3 Results

### 3.1 Demographic and clinical characteristics

The baseline characteristics of enrolled subjects were presented in Table 2. The study included 84 patients (50 males and 34 females) with a mean age of 48.05 ± 15.66 years, as well as 216 healthy controls (126 males and 90 females) with a mean age of 47.22 ± 5.89 years. No significant differences were observed between the cases and controls in terms of age, gender, smoking or alcohol use (*P* > 0.05). Regarding UC lesion location, extensive colitis (E3) (52/84, 61.9 %) was more frequent than distal colitis (E1 + E2) (32/84, 38.1 %) of the patients. For UC severity, 18 patients (21.43 %) were in clinical remission, 21 (25 %) had mild symptoms, 24 (28.57 %) had intermediate symptoms, and 21 (25 %) had severe symptoms, respectively.

**Table 2:** Characteristics of the patient and control populations.

Characteristics	Case (N = 84)	Control (N = 216)	*P-value*
**Age (years)**	48.05 ± 15.66	47.22 ± 5.89	0.636
**Gender (%)**			
Male	50 (59.52)	126 (58.33)	0.851
Female	34 (40.48)	90 (41.67)	
**Smoking (%)**			
Yes	13 (15.48)	35 (16.20)	0.877
No	71 (84.52)	181 (83.80)	
**Alcohol (%)**			
Yes	15 (17.86)	47 (21.76)	0.454
No	69 (82.14)	169 (78.24)	
**Lesion location (%)**			
Distal colitis (E1 + E2)	32 (38.10)		
Extensive colitis (E3)	52 (61.90)		
**Severity (%)**			
Clinical remission	18 (21.43)		
Mild	21 (25.00)		
Intermediate	24 (28.57)		
Severe	21 (25.00)		

### 3.2 Associations of MIR155HG polymorphisms with UC risk

All samples from both the case and control groups were successfully genotyped for the four *MIR155HG* SNPs. Genotype distributions of the four SNPs in the control groups were consistent with HWE (*P* > 0.05). The allele and genotype distributions of *MIR155HG* polymorphisms in UC cases and healthy controls were presented in Table 3. Overall, the rs2282471 polymorphism were observed to have significant differences of allelic and genotypic distributions between case and control groups. Allele genetic and logistic regression analyses revealed that the T minor allele (*P* = 0.030, OR = 0.62, 95 % CI = 0.41–0.96), the TT homozygote genotype (*P* = 0.012, OR = 0.11, 95 % CI = 0.02–0.87), and the recessive model TT vs CT + CC (*P* = 0.016, OR = 0.13, 95 % CI = 0.02–0.95) of rs2282471 was associated with a reduced risk of UC, respectively. Stratified analysis of case and control groups showed a significant association between the rs2282471 polymorphism (TT + CT vs CC) and reduced UC risk in males (*P* = 0.009, OR = 0.39, 95 % CI = 0.19–0.80) (Table 4). Regarding UC patients, the significant associations were observed only in the rs2282471 polymorphism among males. The CT heterozygote genotype (*P* = 0.004, OR = 0.10, 95 % CI = 0.26–0.66) and TT + CT vs CC (*P* = 0.002, OR = 0.25, 95 % CI = 0.10–0.62) were both associated with a reduced risk of UC (Table 5).

**Table 3:** The allele and genotype distribution of *MIR155HG* polymorphisms in UC cases and healthy controls.

SNP	Allele or genotype	Case	Control	*P*-value	OR (95 % *CI)*
		N (%)	N (%)		
**rs1893650**					
	C	36 (21.43)	83 (19.21)	0.541	1.15 (0.74–1.78)
	T	132 (78.57)	349 (80.79)		
	TT	52 (61.90)	141 (65.28)		1.00 (Reference)
	CT	28 (33.33)	67 (31.02)	0.652	1.13 (0.66–1.95)
	CC	4 (4.76)	8 (3.70)	0.630	1.36 (0.39–4.69)
Dominant	CC + CT vs TT			0.584	1.16 (0.69–1.95)
Recessive	CC vs CT + TT			0.675	1.30 (0.38–4.44)
**rs2282471**					
	T	34 (20.24)	125 (28.94)	**0.030**	**0.62 (0.41–0.96)**
	C	134 (79.76)	307 (71.06)		
	CC	51 (60.71)	110 (50.93)		1.00 (Reference)
	CT	32 (38.10)	87 (40.28)	0.390	0.79 (0.47–1.34)
	TT	1 (1.19)	19 (8.80)	**0.012∗∗**	**0.11 (0.02–0.87)**
Dominant	TT + CT vs CC			0.127	0.67 (0.40–1.12)
Recessive	TT vs CT + CC			**0.016∗**	**0.13 (0.02–0.95)**
**rs2829803**					
	A	36 (21.43)	86 (19.91)	0.678	1.10 (0.71–1.70)
	G	132 (78.57)	346 (80.09)		
	GG	52 (61.90)	138 (63.89)		1.00 (Reference)
	AG	28 (33.33)	70 (32.41)	0.829	1.06 (0.62–1.83)
	AA	4 (4.76)	8 (3.70)	0.654	1.33 (0.38–4.59)
Dominant	AA + AG vs GG			0.749	0.92 (0.55–1.55)
Recessive	AA vs GG + AG			0.675	1.30 (0.38–4.44)
**rs2829806**					
	G	36 (21.43)	83 (19.21)	0.541	1.15 (0.74–1.78)
	T	132 (78.57)	349 (80.79)		
	TT	52 (61.90)	141 (65.28)		1.00 (Reference)
	GT	28 (33.33)	67 (31.02)	0.652	1.13 (0.66–1.95)
	GG	4 (4.76)	8 (3.70)	0.630	1.36 (0.39–4.69)
Dominant	GT + GG vs TT			0.584	1.16 (0.69–1.95)
Recessive	GG vs GT + TT			0.675	1.30 (0.38–4.44)

Note: Bold values indicate statistical significance (*P* < 0.05). OR: odds ratio; 95 % CI: 95 % confidence interval.

∗∗P < 0.0125 (0.05/4), ∗P < 0.0167 (0.05/3), indicates statistical significance for Holm-Bonferroni correction.

**Table 4:** Subgroup analyses of *MIR155HG* polymorphisms between UC cases and healthy controls.

SNP variable	N (Case/control)			*P-value*; OR (95 % CI)			
**rs1893650**	**TT**	**CT**	**CC**	**CT vs TT**	**CC vs TT**	**CC vs TT** + **CT**	**CC** + **CTvs TT**
Gender							
Male	28/84	19/36	3/6	^a^0.197; 1.58 (0.79–3.19)	^b^0.692; 1.50 (0.35–6.40)	^b^0.715; 1.28 (0.31–5.32)	^a^0.185; 1.57 (0.80–3.07)
Female	24/57	9/31	1/2	^a^0.407; 0.69 (0.29–1.67)	^b^1.000; 1.19(0.10–13.73)	^b^1.000; 1.33 (0.12–15.20)	^a^0.449; 0.72 (0.31–1.69)
Age (years)							
<40	15/12	6/11	3/1	^a^0.190; 0.44 (0.12–1.52)	^b^0.621; 2.4 (0.22–26.12)	^b^0.609; 3.29 (0.32–34.08)	^a^0.383; 0.6 (0.19–1.90)
>40	37/129	22/56	1/7	^a^0.314; 1.37 (0.74–2.53)	^b^1.000; 0.50 (0.06–4.18)	^b^0.684; 0.45 (0.05–3.72)	^a^0.431; 0.27 (0.70–2.32)
Smoking							
Yes	6/24	6/10	1/3	^b^0.292; 2.4 (0.62–9.27)	^b^1.000; 1.33 (0.12–15.20)	^b^1.000; 0.94 (0.09–9.97)	^a^0.236; 2.15 (0.60–7.77)
No	46/117	22/57	3/5	^a^0.952; 0.98 (0.54–1.79)	^b^0.690; 1.53 (0.35–6.65)	^b^0.691; 1.54 (0.36–6.60)	^a^0.931; 1.03 (0.58–1.82)
Alcohol							
Yes	10/33	4/12	1/2	^b^1.000; 1.1 (0.29–4.18)	^b^1.000; 1.65 (0.14–20.15)	^b^1.000; 1.61 (0.14–19.08)	^b^1.000; 1.18 (0.34–4.08)
No	42/108	24/55	3/6	^a^0.706; 1.12 (0.62–2.04)	^b^0.713; 1.29 (0.31–5.38)	^b^0.721; 1.23 (0.30–5.08)	^a^0.660; 1.14 (0.64–2.03)

**rs2282471**	**CC**	**CT**	**TT**	**CT vs CC**	**TT vs CC**	**TT vs CT** + **CC**	**TT** + **CT vs CC**
Gender							
Male	37/66	13/45	0/15	^a^0.075; 0.52 (0.25–1.08)	**^b^0.003∗∗**	^b^**0.007∗**	^a^**0.009; 0.39 (0.19**–**0.80)**
Female	14/44	19/42	1/4	^a^0.393; 1.42 (0.63–3.20)	**^b^**1.000; 0.79 (0.08–7.62)	^b^1.000; 0.65 (0.07–6.05)	^a^0.443; 1.37 (0.62–3.04)
Age (years)							
<40	15/14	9/7	0/3	^a^0.771; 1.2 (0.35–4.09)	**^b^**0.229	^b^0.234	^a^0.768; 0.84 (0.26–2.67)
>40	36/96	23/80	1/16	^a^0.386; 0.77 (0.42–1.40)	**^b^**0.072; 0.17 (0.02–1.30)	^b^0.082; 0.19 (0.02–1.44)	^a^0.176; 0.67 (0.37–1.20)
Smoking							
Yes	9/22	4/9	0/6	^b^1.000; 1.09 (0.27–4.45)	**^b^**0.302	^b^0.319	^b^0.742; 0.65 (0.17–2.51)
No	42/88	28/78	1/13	^a^0.324; 0.75 (0.43–1.33)	**^b^**0.065; 0.16 (0.02–1.27)	^b^0.122; 0.18 (0.02–1.42)	^a^0.154; 0.67 (0.38–1.17)
Alcohol							
Yes	10/24	5/16	0/7	^a^0.650; 0.75 (0.22–2.61)	**^b^**0.164	^b^0.180	^a^0.290; 0.52 (0.15–1.76)
No	41/86	27/71	1/12	^a^0.443; 0.80 (0.45–1.42)	**^b^**0.108; 0.17 (0.02–1.39)	^b^0.116; 0.19 (0.02–1.51)	^a^0.231; 0.71 (0.40–1.25)

**rs2829803**	**GG**	**GA**	**AA**	**GA vs GG**	**AA vs GG**	**AA vs GG** + **GA**	**AA + GA vs GG**
Gender							
Male	28/83	19/37	3/6	^a^0.238; 1.52 (0.76–3.06)	**^b^**0.694; 1.48 (0.35–6.32)	^b^0.715; 1.28 (0.31–5.32)	^a^0.221; 1.52 (0.78–2.96)
Female	24/55	9/33	1/2	^a^0.293; 0.62 (0.26–1.51)	**^b^**1.000; 1.15 (0.10–13.25)	^b^1.000; 1.33 (0.12–15.2)	^a^0.328; 0.65 (0.28–1.53)
Age (years)							
<40	15/12	6/11	3/1	^a^0.190; 0.44 (0.12–1.52)	**^b^**0.621; 2.40 (0.22–26.12)	^b^0.609; 3.29 (0.32–34.08)	^a^0.383; 0.60 (0.19–1.90)
>40	37/126	22/59	1/7	^a^0.443; 1.27 (0.69–2.34)	**^b^**0.686; 0.49 (0.06–4.08)	^b^0.684; 0.45 (0.05–3.72)	^a^0.576; 1.19 (0.65–2.16)
Smoking							
Yes	6/24	6/10	1/3	^b^0.292; 2.40 (0.62–9.27)	**^b^**1.000; 1.33 (0.12–15.2)	^b^1.000; 0.94 (0.09–9.97)	^a^0.236; 2.15 (0.60–7.77)
No	46/114	22/60	3/5	^a^0.753; 0.91 (0.50–1.65)	^b^0.693; 1.49 (0.34–6.48)	^b^0.691; 1.54 (0.36–6.60)	^a^0.870; 0.95 (0.54–1.69)
Alcohol							
Yes	10/32	4/13	1/2	^b^1.000; 0.98 (0.26–3.71)	^b^1.000; 1.60 (0.13–19.56)	^b^1.000; 1.61 (0.14–19.08)	^b^1.000; 1.07 (0.31–3.67)
No	42/106	24/57	3/6	^a^0.842; 1.06 (0.59–1.93)	^b^0.716; 1.26 (0.30–5.28)	^b^0.721; 1.23 (0.30–5.08)	^a^0.789; 1.08 (0.61–1.92)

**rs2829806**	**TT**	**GT**	**GG**	**GT vs TT**	**GG vs TT**	**GG vs TT** + **GT**	**GG** + **GT vs TT**
Gender							
Male	28/84	19/36	3/6	^a^0.197; 1.58 (0.77–3.18)	^b^0.692; 1.50 (0.35–6.43)	^b^0.715; 1.28 (0.31–5.37)	^a^0.185; 1.57 (0.84–3.06)
Female	24/57	9/31	1/2	^a^0.407; 0.69 (0.30–1.57)	^b^1.000; 1.19 (0.10–13.74)	^b^1.000; 1.33 (0.12–15.19)	^a^0.449; 0.72 (0.35–1.54)
Age (years)							
<40	15/12	6/11	3/1	^a^0.190; 0.44 (0.12–1.54)	^b^0.621; 2.40 (0.22–28.12)	^b^0.609; 3.29 (0.35–31.74)	^a^0.383; 0.60 (0.19–1.89)
>40	37/129	22/56	1/7	^a^0.314; 1.37 (0.74–2.53)	^b^1.000; 0.50 (0.06–4.18)	^b^0.684; 0.45 (0.05–3.72)	^a^0.431; 1.27 (0.70–2.32)
Smoking							
Yes	6/24	6/10	1/3	^b^0.292; 2.40 (0.62–9.27)	^b^1.000; 1.33 (0.12–15.20)	^b^1.000; 0.94 (0.09–9.97)	^a^0.236; 2.15 (0.60–7.77)
No	46/117	22/57	3/5	^a^0.952; 0.98 (0.54–1.79)	^b^0.690; 1.53 (0.35–6.65)	^b^0.692; 1.54 (0.36–6.60)	^a^0.931; 1.03 (0.58–1.82)
Alcohol							
Yes	10/33	4/12	1/2	^b^1.000; 1.10 (0.29–4.18)	^b^1.000; 1.65 (0.14–20.15)	^b^1.000; 1.61 (0.14–19.08)	^b^1.000; 1.18 (0.34–4.08)
No	42/108	24/55	3/6	^a^0.705; 1.12 (0.62–2.04)	^b^0.713; 1.29 (0.31–5.38)	^b^0.721; 1.23 (0.30–5.08)	^a^0.660; 1.14 (0.64–2.03)

Bold values indicate statistical significance (*P* < 0.05). N: numbers; OR: odds ratio; 95 % CI: 95 % confidence interval.

^a^P values were calculated by Pearson chi-square test.

^b^P values were calculated by Fisher's exact test.

∗∗P < 0.00625 (0.05/8), ∗P < 0.00714 (0.05/7), indicates statistical significance for Holm-Bonferroni correction.

**Table 5:** Characterization of clinical features linked to *MIR155HG* SNP genotypes in UC patients.

SNP	Gender (Male/Female)			Age (years) (<40/>40)			Smoking (No/Yes)			Alcohol (No/Yes)		
	Male	Female	*P*; OR (95 % *CI*)	<40	>40	*P*; OR (95 % *CI*)	Yes	No	*P*; OR (95 % *CI*)	Yes	No	*P*; OR (95 % *CI*)
**rs1893650**												
TT	28	24	1.000 (Reference)	15	37	1.000 (Reference)	6	46	1.000 (Reference)	10	42	1.000 (Reference)
CT	19	9	**^a^**0.225; 1.81 (0.69–4.74)	6	22	0.472**^a^**; 0.67 (0.23–1.99)	6	22	**^a^**0.326; 0.48 (0.14–1.65)	4	24	**^b^**0.760; 1.43 (0.40–5.05)
CC	3	1	**^b^**0.620; 2.57 (0.25–26.37)	3	1	**^b^**0.093; 7.40 (0.71–76.92)	1	3	**^b^**0.423; 0.43 (0.04–4.39)	1	3	**^b^**1.000 0; 0.71 (0.67–7.61)
CC vs TT + CT	3/47	1/33	**^b^**0.644; 2.11 (0.21–21.15)	3/21	1/60	**^b^**0.068; 8.43 (0.83–85.54)	1/12	3/68	**^b^**0.496; 0.53 (0.05–5.52)	1/14	3/66	**^b^**0.542; 0.62 (0.06–6.38)
CC + CT vs TT	22/28	10/24	**^a^**0.177; 1.89 (0.75–4.76)	9/15	23/37	**^a^**0.943; 0.97 (0.36–2.56)	7/6	25/46	**^b^**0.227; 0.47 (0.14–1.54)	5/10	27/42	**^a^**0.675; 1.29 (0.40–4.17)
**rs2282471**												
CC	37	14	1.000 (Reference)	15	36	1.000 (Reference)	9	42	1.000 (Reference)	10	41	1.000 (Reference)
CT	13	19	^**a**^**0.004∗; 0.10 (0.26–0.66)**	9	23	**^a^**0.900; 0.94 (0.35–2.50)	4	28	**^a^**0.530; 1.50 (0.42–5.35)	5	27	**^a^**0.646; 1.32 (0.41–4.28)
TT	0	1	**^b^**0.288	0	1	**^b^**1.000	0	1	**^b^**1.000	0	1	**^b^**1.000
TT vs CT + CC	0/50	1/33	**^b^**0.222	0/24	1/59	**^b^**1.000	0/13	1/70	**^b^**1.000	0/15	1/68	**^b^**1.000
TT + CT vs CC	13/37	20/14	^**a**^**0.002∗∗;0.25 (0.10–0.62)**	9/15	24/36	**^a^**0.832; 0.90 (0.34–2.39)	4/9	29/42	**^a^**0.494; 1.55 (0.44–5.53)	5/10	28/41	**^a^**0.602; 1.37 (0.42–4.43)
**rs2829803**												
GG	28	24	1.000 (Reference)	15	37	1.000 (Reference)	6	46	1.000 (Reference)	10	42	1.000 (Reference)
GA	19	9	**^a^**0.225; 1.81 (0.69–4.74)	6	22	**^b^**0.472; 0.67 (0.23–1.99)	6	22	**^b^**0.326; 0.48 (0.14–1.65)	4	24	**^b^**0.760; 1.43 (0.40–5.05)
AA	3	1	**^b^**0.620; 2.57 (0.25–26.37)	3	1	**^b^**0.093; 7.40 (0.71–76.92)	1	3	**^b^**0.423; 0.39 (0.04–4.39)	1	3	**^b^**1.000; 0.71 (0.07–7.61)
AA vs GG + GA	3/47	1/33	**^b^**0.644; 2.11 (0.21–21.15)	3/21	1/59	**^b^**0.068; 8.43 (0.83–85.54)	1/12	3/68	**^b^**0.496; 2.57 (0.55–5.52)	1/14	3/66	**^b^**0.552; 2.57 (0.06–6.58)
AA + GA vs GG	22/28	10/24	**^a^**0.177; 1.89 (0.75–4.76)	9/15	23/37	**^b^**1.000; 1.00 (0.03–29.81)	7/6	25/46	**^a^**0.227; 0.47 (0.14–1.54)	5/10	27/42	**^a^**0.675; 1.29 (0.40–4.17)
**rs2829806**												
TT	28	24	1.000 (Reference)	15	37	1.000 (Reference)	6	46	1.000 (Reference)	10	42	1.000 (Reference)
GT	19	9	**^a^**0.225; 1.48 (0.77–2.86)	6	22	**^b^**0.472; 0.77 (0.36–1.63)	6	22	**^b^**0.326; 0.65 (0.33–1.25)	4	24	**^b^**0.760; 1.27 (0.52–3.09)
GG	3	1	**^b^**0.620; 2.11 (0.21–21.15)	3	1	**^b^**0.093; 7.40 (0.71–76.92)	1	3	**^b^**0.423; 0.39 (0.04–4.39)	1	3	**^b^**1.000; 0.71 (0.07–7.61)
GG vs TT + GT	3/47	1/33	**^b^**0.644; 2.57 (0.25–26.37)	3/21	1/59	**^b^**0.068; 8.43 (0.83–85.54)	1/12	3/68	**^b^**0.496; 0.53 (0.05–5.52)	1/14	3/66	**^b^**0.552; 0.64 (0.06–6.58)
GG + GT vs TT	22/28	10/24	**^a^**0.177; 1.89 (0.75–4.76)	9/15	23/37	**^a^**0.943; 0.47 (0.36–2.56)	7/6	25/46	**^b^**0.227; 0.47 (0.14–1.54)	5/10	27/42	**^a^**0.675; 1.29 (0.40–4.17)

Bold values indicate statistical significance (*P* < 0.05), OR: odds ratio, 95 % CI: 95 % confidence interval.

^a^*P* values were calculated by Pearson chi-square test.

^b^*P* values were calculated by Fisher's exact test.

∗∗*P* < 0.0125 (0.05/4), ∗*P* < 0.0167 (0.05/3), indicates statistical significance for Holm-Bonferroni correction.

### 3.3 Haplotype analysis of MIR155HG polymorphisms

**Fig. 1:**
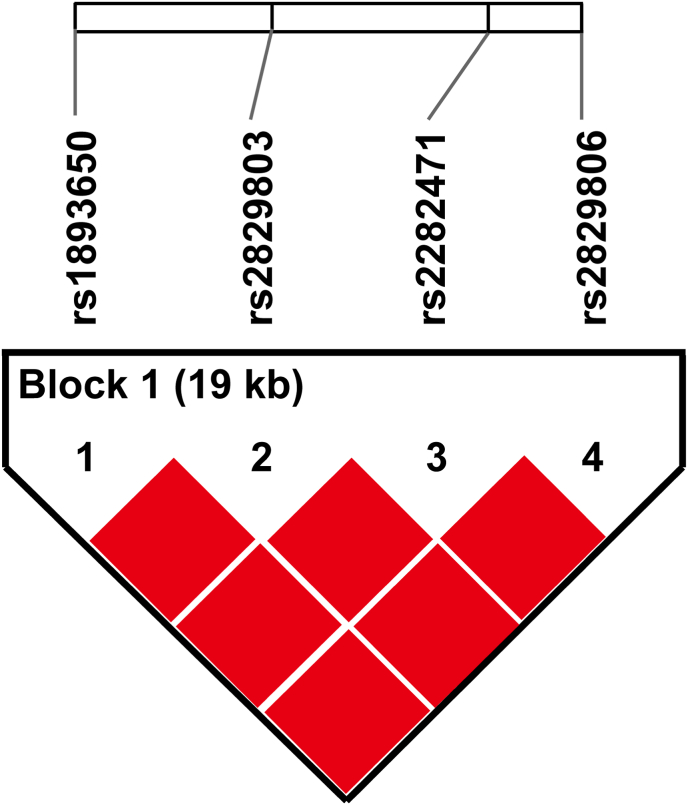
Linkage disequilibrium analysis of the SNPs in *MIR155HG gene*. The linkage disequilibrium (LD) of four *MIR155HG* SNPs was determined using Haploview software 4.2. These four *MIR155HG* SNPs formed a single haplotype block. LD: Linkage disequilibrium.

The haplotype association analysis revealed that rs1893650, rs2829803, rs2282471, and rs2829806 of the *MIR155HG* formed a single haplotype block, suggesting a strong LD among these four SNPs loci (Fig. 1). Three haplotypes were identified by LD analysis, among which the TGTT haplotype was associated with a reduced risk of UC (*P* = 0.035, OR = 0.63, 95 % CI = 0.41–0.97) (Table 6).

**Table 6:** The Haplotype distribution of *MIR155HG* polymorphisms between UC cases and healthy controls.

Haplotype	Haplotype Frequency		*P-*value	OR (95 % *CI)*
	Case	Control		
TGCT	0.58	0.51	0.126	1.32 (0.92–1.90)
TGTT	0.20	0.29	**0.035**	**0.63 (0.41–0.97)**
CACG	0.21	0.19	0.541	1.15 (0.74–1.78)

*Note*: Bold values indicate statistical significance (*P* < 0.05). OR: odds ratio; 95 % CI: 95 % confidence interval.

### 3.4 Associations between MIR155HG polymorphisms and clinical characteristics of UC

We further evaluated the association between *MIR155HG* polymorphisms and the clinical characteristics of the UC cases. No significant associations were observed between *MIR155HG* polymorphisms and either the severity or the lesion location of UC (Table 7).

**Table 7:** The associations between *MIR155HG* polymorphisms and clinical characteristics of ulcerative colitis.

SNP	Severity of UC		Lesion location of UC	
	Severe/Remission + Mild + Intermediate	*P*-value; OR (95 % *CI*)	E3/E1+E2	*P*-value; OR (95 % *CI*)
**rs1893650**				
TT	11/41	1.000 (reference)	34/18	1.000 (reference)
CT	9/19	0.279; 1.77 (0.63–4.97)	15/13	0.427; 0.61 (0.24–1.56)
CC	1/3	0.857; 1.24 (0.12–13.15)	3/1	0.876; 1.59 (0.15–16.39)
CC + CT	10/22	0.299; 1.69 (0.62–4.61)	18/14	0.545; 0.68 (0.28–1.68)
**rs2282471**				
CC	11/40	1.000 (reference)	29/22	1.000 (reference)
CT	9/23	0.497; 1.42 (0.51–3.94)	22/10	0.395; 1.67 (0.66–4.23)
TT	0/1	0.601	1/0	0.875
TT + CT	9/24	0.549; 1.36 (0.49–3.77)	23/10	0.341; 1.74 (0.69–4.41)
**rs2829803**				
GG	11/41	1.000 (reference)	34/18	1.000 (reference)
GA	9/19	0.279; 1.77 (0.63–4.97)	15/13	0.427; 0.61 (0.24–1.56)
AA	1/3	0.857; 1.24 (0.12–13.15)	3/1	0.876; 1.59 (0.15–16.39)
AA + GA	10/22	0.299; 1.69 (0.62–4.61)	18/14	0.545; 0.68 (0.28–1.68)
**rs2829806**				
TT	11/41	1.000 (reference)	34/18	1.000 (reference)
GT	9/19	0.279; 1.77 (0.63–4.97)	15/13	0.427; 0.61 (0.24–1.56)
GG	1/3	0.857; 1.24 (0.12–13.15)	3/1	0.876; 1.59 (0.15–16.39)
GG + GT	10/22	0.299; 1.69 (0.62–4.61)	18/14	0.545; 0.68 (0.28–1.68)

*Note*: OR: odds ratio; 95 % CI: 95 % confidence interval.

### 3.5 The function prediction of the MIR155HG polymorphisms

**Fig. 2:**
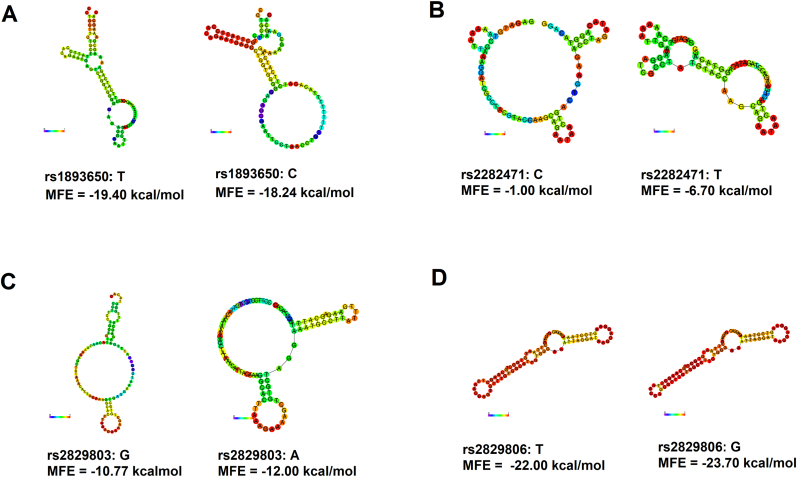
In silico prediction of secondary structures and MFE changes corresponding to *MIR155HG* SNPs using RNAfold. The MFE changed from −1.00 kcal/mol to −6.70 kcal/mol due to the rs2282471 (C > T) variant, indicating a stronger binding affinity of miRNAs to the T allele (B). In contrast, the changes in MFE values for rs1893650 (A), rs2829803 (C), and rs2829806 (D) were not significant before and after the base-pair substitutions. MFE: minimum free energy.

The centroid secondary structures of *MIR155HG* polymorphisms were shown in Fig. 2. The RNAfold predicted that the rs2282471C > T substitution significantly altered the centroid secondary structure of miR-155, with the MFE decreasing from −1.00 kcal/mol to −6.70 kcal/mol. This suggested that the T allele had a higher binding affinity to miRNAs than the C allele. In contrast, the changes in MFE value for other three SNPs (rs1893650, rs2829803, and rs2829806) were not significant before and after the base-pair substitutions.

## 4 Discussion

The current study investigated the association between polymorphisms in the *MIR155HG* gene and susceptibility to UC in a Chinese Han population. Our findings suggested that the rs2282471 polymorphism was associated with a decreased risk of UC among Han Chinese individuals. Specifically, the minor T allele, the homozygous TT genotype, and the recessive genetic model (TT vs. CT + CC) of the rs2282471 polymorphism were identified as protective factors against UC development. Stratified analysis revealed that these protective effects were particularly obvious in male patients with CT genotype and TT + CT vs CC of rs2282471 polymorphisms. The haplotype analysis demonstrated strong linkage disequilibrium among the four studied SNPs, and identified a specific haplotype, TGTT, that was also associated with a reduced UC risk. Furthermore, we found rs2282471 may influence the secondary structure of miR-155, of which T allele had a higher binding affinity to miRNAs than the C allele. However, no significant correlations were observed between these polymorphisms and clinical features such as disease severity or lesion location.

Genome-wide association studies have identified more than 240 genetic loci associated with IBD [22,23]. These loci collectively contribute to understanding the complex genetic architecture underlying IBD and provide potential targets for developing novel diagnostic tools and therapeutic interventions. MiR-155, encoded by the *MIR155HG gene* located on Chromosome 21, belongs to the miRNA family and exerts post-transcriptional regulation of gene expression by binding to the 3′UTR of target mRNA. MiR-155 is widely recognized as a key modulator of both innate and adaptive immunity, participating in the activation of T helper cells (Th1, Th17), B cells, and macrophages, as well as the production of pro-inflammatory cytokines (e.g., TNF-α, IFN-γ, IL-17) [24]. Abnormal expression of MiR-155 has been observed in several inflammatory autoimmune disorders, including type 1 diabetes, rheumatoid arthritis, systemic lupus erythematosus, multiple sclerosis, systemic sclerosis, and IBD [8]. Particularly, miR-155 expression is significantly up-regulated in the inflamed intestinal mucosa of IBD patients, where it promotes the release of pro-inflammatory cytokines such as TNF-α [25]. Moreover, previous studies have indicated that serum miR-155 levels were increased in UC patients compared to healthy controls [26,27].

Given pivotal role of miR-155 in immunopathogenesis of UC, genetic variations in *MIR155HG* gene that alter its expression or function may be plausible candidates for influencing the UC susceptibility. Polymorphisms in the *MIR155HG* gene can affect the transcription efficiency or stability of the miR-155 precursor, thereby regulating the expression level of mature miR-155. Our findings indicated that the T allele, homozygous TT genotype, and the recessive genetic model (TT vs. CT + CC) of the rs2282471 polymorphism were protective. This aligned with the well-established role of miR-155 in regulating immune responses, and suggested that this genetic variant may lead to a reduction in the release of pro-inflammatory factors and alleviate intestinal mucosal damage, and thereby reducing the likelihood of developing UC. These results were consistent with accumulating evidence that miRNA polymorphisms were involved in the pathogenesis of autoimmune and chronic inflammatory diseases [8,18].

Interestingly, the significant association between rs2282471 polymorphisms (CT and TT + CT vs. CC) and a reduced risk of UC was exclusively observed in male patients in this study. The incidence and clinical course of UC may differ between sexes, which were potentially influenced by hormonal and genetic factors [28]. Although the underlying mechanism for this sex-specific association remains unclear, it underscores the complexity of UC etiology and highlights the necessity of considering sex as a biological variable in genetic association studies. Future investigations with larger sample sizes are therefore warranted to validate this observation and explore its basis.

Haplotype analysis integrates the combined effects of multiple SNPs within a gene, which can better reflect the cumulative impact of genetic variations on disease risk compared to single SNP analysis [29].The identification of a protective effect of the haplotype (TGTT) formed by the four SNPs (rs1893650, rs2829803, rs2282471, rs2829806) in the present study suggested that the combined variation of these four SNPs may synergistically modulate miR-155 expression, thereby further influencing UC pathogenesis. Particularly, the TGTT haplotype harbored the protective T allele of rs2282471, which provided additional support for the potential role of rs2282471 in UC susceptibility.

In this study, we observed that the rs2282471 C > T substitution altered the secondary structure of miR-155. Previous studies have reported that such structural changes can affect the stability and translation of mRNA. Therefore, rs2282471 might regulate the translation and subsequent Tregs’ function by this way. This finding further established a potential association between genetic variation in miR-155 and the functional modulation of Tregs, which were key players in maintaining immune homeostasis. It also provided a hypothesis for the rs2282471 polymorphism could contribute to the pathogenesis of immune-related disorders, including IBDs.

In this study, no significant associations were observed between *MIR155HG* polymorphisms and clinical characteristics of UC, including lesion location (extensive colitis vs. distal colitis) and disease severity (remission, mild, moderate, severe). This finding may be attributed to the fact that *MIR155HG* polymorphisms may primarily influence the susceptibility to UC rather than the disease's progression or phenotypic manifestations. However, this assumption should be interpreted with caution due to the small sample size of UC patients in our study, which may have limited the statistical power to detect associations.

Our study had several limitations. First, the sample size, particularly that of the UC cohort, was relatively small, which may constrain the statistical power for stratified analyses (e.g., by gender, smoking, or alcohol consumption) and for the detection of associations with weaker effects or clinical subtypes. Second, the study population was exclusively of Chinese Han ethnicity, therefore the generalizability of our findings to other ethnic groups requires validation duo to inherent differences in genetic backgrounds and environmental factors (e.g., dietary patterns and lifestyle behaviors) across populations. Third, this is an exploratory genetic association study, and the functional implications of the rs2282471 polymorphism on miR-155 expression and its biological activity remain unelucidated. Nonetheless, this study still provided evidence that *MIR155HG* polymorphisms were associated with UC risk in the Chinese Han population, which laid a foundation for further exploring the immunogenetic mechanisms underlying UC pathogenesis.

In conclusion, this study demonstrated that the rs2282471 polymorphism of *MIR155HG* were associated with a reduced risk of UC in the Chinese Han population. To our knowledge, this is the first study to evaluate the relationship between *MIR155HG* gene SNPs and ulcerative colitis in Han Chinese population. These findings suggested that *MIR155HG* might be a potential genetic susceptibility locus for UC and offered novel insights for understanding the immunogenetic mechanisms of the disease. Further large-scale, multi-ethnic studies, coupled with functional experiments, are essential to validate these associations and to unravel the precise biological mechanisms through *MIR155HG* variants modulate intestinal inflammation.

## CRediT authorship contribution statement

**Chao Xu:** Writing – original draft, Methodology, Investigation, Formal analysis, Data curation, Conceptualization. **Beibei Zhou:** Methodology, Investigation, Formal analysis. **Fan Bai:** Software, Methodology, Investigation, Data curation. **Hang Sun:** Methodology, Investigation. **Cuiping Zhang:** Investigation, Data curation. **Zhenlun Wu:** Investigation. **Jiachuan Hou:** Investigation. **Jinjing Xie:** Investigation. **Yanbin Wei:** Investigation. **Libin Pan:** Investigation. **Ruiqi Yang:** Investigation. **Hongjie Dong:** Investigation. **Guihua Zhao:** Investigation. **Jingyu Yang:** Supervision. **Jianwei Zhou:** Writing – review & editing, Resources, Investigation, Data curation. **Ruili Wu:** Writing – review & editing, Resources, Investigation. **Kun Yin:** Writing – review & editing, Supervision, Project administration.

## Funding

This study was supported by the Medicine and Health Science Technology Plan of Shandong Province (202312051358); the Taishan Scholars Project of Shandong Province (tsqn202103186); Joint Project of Medical Staff Scientific and Technological Innovation Program of Shandong Province (SDYWZGKCJHLH202423); Joint Innovation Team for Clinical & Basic Research (202407); the Innovation Project of Shandong Academy of Medical Sciences.

## Declaration of competing interest

The authors declare that they have no known competing financial interests or personal relationships that could have appeared to influence the work reported in this paper.
